# Loot

**Published:** 1871-01

**Authors:** 


### Loot.

***Dangers of Chloral.***

Dr. R. C. Shettle, physician to the Royal Berkshire Hospital, in the course of an address before the Pathological Society of Reading, after referring to Dr. Richardson's statement that under the influence of chloral formiate of soda is formed, and the coagulating power of the blood is much diminished, adds that "it is very evident that not only is passive hæmorrhage from an open vessel favored by the administration of chloral, but from the tendency which exists from the continuous administration of the drug to produce decomposition of the blood, a small loss of blood would be very possibly followed by serious symptoms. Moreover, it can scarcely be a question that the tolerance of the drug is much greater in some individuals than in others; and possibly the same person at different times might bear its administration very differently." If these views be correct, much caution would seem to be called for in using chloral in midwifery cases.—*Med. Gazette.*

***White India Rubber.***

White India Rubber, as used in nursing-bottles for infants, contains an active poisonous ingredient, causing sore mouth, eruption, and even death in some cases; its sale is, on this account, prohibited by law in some European countries.

***Improved Test for Grape Sugar.***

Fehling's test fluid, it is well known, is not very permanent, spoiling even in diffused daylight. Lowe recommends the following modification of it, which is equally sensitive, and very stable: Sixteen grammes of pure sulphate of vitriol are dissolved in 64 grammes of water. To this solution 80 cubic centimetres of soda lye, having a specific gravity of 1·34, are added at a time, avoiding any elevation of temperature, until 112 grammes have been mixed; then from 6 to 8 grammes of pure officinal glycerine are poured in till the fluid assumes a beautiful azure blue color. *Lancet.*

***Infallibility.***

Geo. M. Hambright, a correspondent of the *Pharmaceutist*, is "down on" the M.Ds. in an article from which we scissor this, with the single deprecatory remark, that, perhaps, in many of the instances given, the apparent errors were due to bad chirography rather than sheer ignorance. Meanwhile, brethren, mind your Ps and Qs:

"The 'infallibility of M.Ds.' does not only exist in the practice of the healing art, but also in that which is next in importance to a correct diagnosis, viz.: *prescribing*. Scarcely any detailed remarks need be made on this point, to prove how wofully deficient the lower class of M.Ds. are in this very important particular. Prescriptions that are daily filled in this city and surroundings would be more fitting *fac-similes of the exhumed hieroglyphics from the Egyptian catacombs* than an order for physic, in this enlightened age, not to make mention of the incompatibles heaped together as the *healing balm*, in terms and language that indicate that the schoolmaster is not always at home among the M.Ds. who move in aristocratic circles.

"As a rule, intelligence and science center in the large cities, and if the appended specimen-productions of the medical men of this focus are taken as paradigms of learning, what is the intellectual condition of the M.Ds. in the by-places of the State?

"The first example will be found on every nine out of ten prescriptions, where the useful article is ordered.

| | | |
|---|---|---|
| Aqua d*i*stil. | Pulv*o* S*ie*dlitz. | Sac*k* Lactis |
| Pulv. Ip*i*cac: | Tr. Card*o*m*an*. | Plum: Carbo. |
| *By* Carb. Potass, | Oleum *Tilgi*. | Sulph: *E*ather. |
| Tr. Val*i*rian. | *Podiphiline*, | Ol*i*um Lini |
| Chl*ordte* Potass: | Compt. Cath. Pillula, etc. | |

"To this list may be added *aqua fluvialis*, which is frequently prescribed by the A 1 M.Ds. in this city; surely a very disgusting medication, when '*the purity of the Chicago river*' is taken into consideration. This article is probably prescribed by some M.Ds. because they suppose it to be one of the healing remedies of the Materia Medica; and, conceding it to possess medical properties, it *may*, *possibly*, bear, in this respect, a strong resemblance to "*aqua mirabile*," with which very few M.Ds. are acquainted.

"As specimen products of second-class philomathes in medicine, allusion will be made to—

| | | |
|---|---|---|
| Bal: Cop*eve.* | Nitric *Either.* | *Colodien—* |
| Tr. Jal*op.* | Pul: *E*p*i*cac, | Lup*o*lin. |
| Sulphatum Ferri. | Junipero Spir*a*tus. | |
| D*iet.* Act: Plumb. | Tr. Clor*h*ide Ferri. | |

"Presuming to specify third-rate M.Ds.' productions, there will be found—

| | |
|---|---|
| 1 *ons Boulson* K. P*v* | *Syr. Senneca Sneke root.* |
| 1 " *Nither* | *Poder: Kubeabs* |
| ¼ " T*urpentin* | B*iconbonat Soder.* |

"And the doctor who wrote for Isinglass Plaster, which he returned in person, with the statement, 'that he wanted plaster with some body, thick and strong,' and, on being shown the roll of *adhesive plaster*, hurriedly exchanged *the compliment of his recipe* for that article, and left.

"As *finis* to this class the following model is given:

RECIPE—Senega oz. i.
Syrup Scilla "
*Morphia* " Mix.
Dose—teaspoonful 4 to 6 hours.

"All the above examples of 'Infallibility of M.Ds.' are taken from a small file, and when compared with those of larger growth the *magnitude of error* is decidedly appalling, and speaks volumes in favor of a better training of the men of medicine, in the art which they practice."

***Croup and Cubebs.***

Of eight cases of angina treated with cubebs, seven recovered and one died. Thirty-four cases of croup were treated with this drug; thirteen recovered — three without tracheotomy, and ten after the operation had been performed. In twenty of the unsuccessful cases, tracheotomy was resorted to. The patients were from sixteen months to nine years old. Only six were seen in the beginning of the second stage; all these were saved. In twenty-seven cases, the operation was performed immediately after entering. Cubebs was given after the operation apparently with better effect than before.—*Gazette des Hopitaux and Ind. Jour. of Medicine.*

### *Belladonna in the Treatment of Typhoid Fever.*

Dr. B. Kelley, of Dublin, recommends very highly the use of belladonna in treating enteric fever. He does not prescribe it until the prominent symptoms are fully developed. If the patient be an adult, his dose is from twenty to twenty-five drops of the officinal tincture of the British Pharmacopœia every four hours, but must be varied to suit ages and constitutions. He says, "It completely changes the whole character and outward manifestation of the disease. Delirium, coma and subsultus quickly vanish, and are succeeded by calmness and clearness of intellect, by natural sleep, and complete control of all the voluntary muscles. Diarrhœa is checked, and healthy, consistent evacuations are established." He gives its most remarkable power as seen in the suddenness with which patients recover their intellectual faculties, and the full control of the muscles. In stopping its use, it is necessary to do so gradually, to prevent serious consequences. All stimulants he interdicts, in every form and shape, while the patient is under the treatment of belladonna. Care must also be exercised that the patient does not get around too soon, which they are very much inclined to do while under its magical influence, and thus occasion a relapse.—*Med. Indep.*

### *Epidemic Diphtheria and its Treatment.*

According to reports from the state of Ibraila, the epidemic of diphtheritic angina which raged in that town sixteen months ago produced 700 deaths up to the 15th of December last, of whom 298 were men and 402 women. In a population of 30,000, the gravity of the epidemic may be estimated. Perchloride of iron, given conjointly with tonics, seemed to be most efficacious, and cauterization with a concentrated solution of nitrate of silver was found very useful when the pseudo-membranous exudation was localized.—*Med. Press and Circular.*

### *Impotence consequent on Onanism cured by Electro-Magnetism.*

Dr. Finstein, of Ulm, reports a case of this in a man aged 26, who, from the age of 15 to 18, was addicted to onanism, which he relinquished after the latter period by the advice of a companion. At the age of 23 he found himself unable to have an erection. Various remedies were tried in vain, and at last electro-magnetism with the apparatus of Erdman was resorted to, and after fifty-two applications, made twice daily, continued from 10 to 30 minutes, his powers were restored.—*Révue de Thérapeutique*, Sept., 1870.—*Med. News.*

***Illustrations of Quackery.***

Every week brings to California some new phase of charlatanism, proclaimed through the newspapers. The last comer announces himself as "Director of the Homœopathic Healing Institute." He has established "specific healing baths," which produce, to use his own lucid language, "A galvanic magnetic fluidum, which can be impregnated with specific healing powers, in order to communicate the same to the vital forces of the organism. The constant influx of this healing fluidum annihilates the dynamical powers of all disease, causes and animates the organic activities so thoroughly, that all disturbances are equalized, all foreign matter expelled through beneficial perspiration, and all parts of the organism brought back to the normal state of health. How beneficial this new healing method is, may be seen from the fact, that all spinal complaints, paralysis, and deformities, also the hip diseases of children, are cured by it. It eases the pains immediately, and cures the quickest female diseases that originate in loss or corruption of sap," and so forth. We think he hit the mark in the last line. Most of his patients will be troubled with "loss or corruption of sap."—*Pacific Med. and Surg. Jour.*

***Mortality of Foundlings.***

Dr. C. R. Drysdale states (Brit. Med. Jour., Oct. 22, 1870): "In Paris, in 1865, of 4,887 children abandoned younger than one year, 1,516 died under the care of the Foundling Hospital. In fact, the mortality of Parisian children is excessive, since that city loses 39 per cent. of all its children under one year of age annually. There is an immensity of illegitimacy in Paris, which accounts for this great mortality. Society, naturally, cannot expect the mother of an illegitimate child to take much care of it, since it makes it almost impossible for an unmarried woman to get a living for herself, if burdened by an infant. Thus, in a private letter to myself from Dr. Leon Lefort, in June last, he informs me that more than one in four of the births in Paris are illegitimate. In some of the foundling hospitals in France as many as 90 per cent. of the infants confided to their care have died in less than a year. Our foundling hospital in Guilford street, is, of course, like many other London charities, a job; and even Mr. Acton was not able to find out whose children got into that favored spot.

***Treatment of Diarrhœa in Children.***

M. Heller recommends the nitrate of bismuth, in doses of half a drachm to one drachm, in the diarrhœa of infants. At the outset this may be repeated every hour till the looseness of the bowels ceases, which usually happens within twenty-four hours. No ill consequences ever result from its employment.—*Deutches Archiv. f. klin. Med. Band.*

### *Treatment of Cancrum Oris.*

Dr. W. S. Edgar, of St. Louis, invites attention (St. Louis Med. and Surg. Journal, Sept. 1870) to the prophylactic treatment as all important in this disease. When small points of ulceration are discovered on the gums and inner surface of the lips and cheeks, and the face or gums begin to swell, and the mucous membrane becomes puffy and shining around the ulcers, the danger is imminent, and the patient should at once be put on nutritious diet, tonics, and change of air when practicable. Among the ferruginous preparations the liquor ferri iodidi is preferred in these cases and it should be given, particularly in malarial regions, alternately with the citrate of iron and quinia. The local treatment is no less important. Thorough cleansing of the ulcers and mouth twice a a day with the nebulizer, using a weak solution of the chloride of sodium or chlorate of potassa; also the permanganate of potassa to correct the offensive odor of the mouth and stimulate healthy granulation. If any escharotic is indicated, a strong solution of sulphate of copper, xxx grs. to the ounce of water, or paste of permanganate of potassa is preferred to the mineral acids, as less painful and less likely to extend beyond the point desired.—*Med. News.*

### *The Local Treatment of Phthisis Pulmonalis and Diphtheritis by Carbolic Acid.*

Dr. Rothie recommends: Acid carbol. crystal; spirit vini., aa. gr. xv to xxx; aq. distilled, dr. i; tincture of iodine, gr. xv; s. 10 to 20 drops, with one ounce of water, to be inhaled. Of six cases of consumption [caused by pneumonia, tubercles, bronchitis] success ensued four times. In diphtheria the following solution for washing and gargling was found: Acid carbol. crys.; spirit vini., aa. gr. xv; aq. distilled, dr. i; tinc. iodine, gr. vii.—*Memorabil.* —*Indiana Jour.*

### *Means of Distinguishing Real from Apparent Death.*

Dr. Laborde communicated to the French Academy of Medicine (July 26, 1870) an interesting memoir on some of the physical phenomena of life, and on their application to distinguishing real from apparent death. He states that if a polished steel needle be inserted to a sufficient depth into the muscles of an apparently dead subject, and allowed to remain, in generally a short time it loses its polish and becomes oxidized; whilst this oxidation does not occur when the needle is introduced into a dead subject, if the needle be allowed to remain even as long as an hour. This absence of oxidation, with concomitant phenomena, is, according to Dr. L., a constant sign of real death.—*L' Union Medicale.*

**Carbolic Acid Preparation.**

Mr. T. A. Redwin, in a paper read before the British Pharmaceutical Conference, gives the following as advisable proportions in the use of carbolic acid:

As a rule, it is better to dissolve the crystallized carbolic acid (Calvert's) in the proportions of one part by weight of the acid to six of glycerine (carbolate of glycerine.) In this state it can be diluted equally indefinitely.

In general, a dose of carbolic acid is 1 grain in an ounce of water.

As a gargle, 1 or 2 grains to an ounce of water.

As an injection, 1 grain to 4 ounces of water.

As a lotion, 15 grains to an ounce of water.

As an ointment, 30 grains to an ounce of benzoated lard.

As a liniment, 1 grain to 20 of olive oil.

As a plaster, 1 part of carbolic acid to 3 of shellac.

The crystallized carbolic acid to be used as a caustic.

The carbolate of glycerine, as above, should be used in 1 or 2 drop doses.

Antiseptic oil for abscesses, 1 part of acid to 4 of boiled linseed oil.

Antiseptic putty, 6 spoonsful of the antiseptic oil mixed with common whiting.

Aqueous solution of carbolic acid is 1 part of acid to 40 of water (1 ounce of acid to a quart of hot water well agitated and filtered.)

Sick rooms, to disinfect, place a portion of the dissolved acid in a porcelain dish, and float it in a large vessel of hot water.

Disinfecting purposes generally, 1 pound of *crystals* to 6 galls. of water. *Fluid*, 1 part to 80 of water. *Powder*, 1 ounce of crystals with 4 pounds of slaked lime.

For drains, take 1 pound of the fluid carbolic acid to 5 gallons of warm water.

Toothache is often cured with one drop of carbolate of glycerine; and diarrhœa arrested in half an hour with 2 drops.

In all cases of parasitic life it is advisable to commence with very dilute carbolate of glycerine.—*Chemist and Druggist.*

**Treatment of Phthisis with Arsenic Acid.**

The dose must be very small, gradually increasing, and occasionally suspended; the treatment is prophylactic. The first 10 days, .016 of a grain daily; the second 10 days, one or two tablespoonsful of cod liver oil daily; the third 10 days no medicine; then as before. This treatment is to be continued from November to May; besides, the diet must be rich and well regulated.—*Abeille Medicale.—Indiana Jour.*

### *Chloral in Puerperal Convulsions.*

Baron Paul von Sergdewitz, of Basle, communicated to the Obstetrical Society of London a case of violent convulsions in a woman suffering from endocarditis subsequent to delivery, in which chloral at once arrested the fits, after various other remedies had been used in vain.—*British Medical Journal.*

Dr. Milne has also used chloral with success in a case of puerperal convulsions. "The case," he says, "was a purely psychical one, due to the shock of a sudden loud and unexpected sound, and therefore well fitted to test the powers of the medicine, these cases being deemed rather obstinate. On the other hand, there was no albuminuria, and the absence of this complication lessened, in some degree, the seriousness of the case, and increased its amenableness to remedies. The benefit I obtained from the chloral was considerable, although not sufficient, in my estimation, to send one into ecstacies, or impress one with the belief that a novel cure had been brought before us, of unexampled power."—*Edinburgh Medical Journal*, May, 1870.

Dr. X., of Bapaume, communicated to the Imperial Surgical Society (March 23, 1870,) through M. Demarquay, a very interesting case of puerperal eclampsia in a primipara. The convulsions came on during labor, and continued after delivery. All the ordinary remedies having failed to afford relief, chloral was had recourse to, in increasing doses, commencing with four grammes. When the dose reached six grammes, the patient fell into a deep and quiet sleep, which continued for twelve hours. After awakening she had slight attacks, which were relieved by chloral, and complete recovery ensued.—*L'Union Med.*

### *Cure for Toothache.*

Dr. Henry T. Reynolds, of Baltimore, writes to us that "for eighteen months I have been using acetate of lead as a remedy for what Burns calls 'the hell of all diseases'—toothache. I find it to act better than any of the numerous remedies proposed in the books, and in cases in which it is applicable the relief is instantaneous. Let the patient apply from one to three grains to the cavity for a moment or two, then spit it out. It fails in fewer cases than any remedy that I have tried—not more than eight per cent.—*Med. News.*

### *Gastric Douche.*

An apparatus for applying the douche to the stomach is described by Dr. Ploss, of Leipsic. It is indicated in chronic gastric catarrh, poisoning, pyloric stricture, and pyrosis. It has a double channel, but resembles the nasal douche in principle.

***Soil and Disease.***

At the present meeting of the British Association in Liverpool, Dr. Moffatt, of Hawarden, has read an interesting paper on "Geological Systems and Endemic Diseases," showing that the soil has an influence on the composition of the cereal plants grown upon it, and on the diseases to which the inhabitants are subject. The district in which he practices consists geologically of the carboniferous and new red sandstone or Cheshire sandstone systems. Anæmia with goitre is prevalent amongst those living on the carboniferous systems, whilst it is almost unknown amongst those living on the new red sandstone system; and consumption is also more prevalent amongst the inhabitants of the former. Dr. M. has found by analysis that the wheat grown on the soil of the Cheshire sandstone contains the largest quantity of ash, and that there is a larger quantity of phosphoric acid and of oxide of iron in it than in the soils of the carboniferous and millstone grit systems. He has calculated that each inhabitant on the Cheshire sandstone, if he consumes a pound of wheat daily, takes in nearly five grains per day of the sesquioxide of iron more than the inhabitant of the carboniferous system, who seems, therefore, to be subject to anæmia in consequence of the deficiency of iron and phosphoric acid in his food. It is not only in the wheat grown upon the carboniferous system that there is a deficiency in the quantity of oxide of iron and the phosphates, according to Dr. Moffatt, but also in the blood of the animals reared upon it. He stated that sheep were liable to anæmia, which he attributed to sheep-walks being upon trap and limestone hills, in the soil of which there is but little, if any, iron.—*British Med. Jour.*

***Medicated Cigarettes.***

The cigarettes of Cannabis Indica, made by Gremault, of Paris, have been found most efficient in the treatment of affections of the organs of respiration and circulation, no less than in affections of the central and peripheral nervous system. The unpleasant effects which so often follow the internal and subcutaneous use of opium and of Cannabis Indica, are not produced by the cigarette. Their use is recommended (1) in spinal neuroses, chorea, and epilepsy; (2) in neurosis of the sensory nerves, neuralgia of the teeth, branches of the fifth pair, the sciatic nerves; (3) neuroses of the motor nerves, spasm of the throat air passages; (4) affections of the sympathetic nerves, hysteria, and other diseases not attended with plethora, and congestion of the head, heart, or lungs. They are especially useful in asthma, pertussis, spasm of the stomach and intestinal canal, nervous palpitation of the heart, and exert a quieting influence over the whole nervous system.—*Allgemeine Med. Zeit.*—*Med. Bulletin.*

***Embrocation for Inflammation of Breasts.***

| | |
|---|---|
| ℞ Fluid Extract of Aconite, - - | One half ounce; |
| " " Phytolacca, - | One ounce; |
| Iodide Potassium, - - - | One drachm; |
| Warm Water, - - - | One pint. |

Wet linen compresses, and apply constantly.

No application with which I am acquainted will so surely prevent tumors of the mammæ from taking on the suppuration process. Even if suppuration have taken place, it resolves the indurations and relieves pain.—*Med. Bulletin.*

***The Pool of Siloam.***

The miraculous efficacy of the Pool of Siloam, as recorded in the Scriptures, is familiar to us all, but its modern condition appears as fraught with danger as was its ancient with the power of healing. Speaking of a fatal case of enteric fever, the surgeon of H.M.S. *Endymion*, Dr. Alex. Fisher, says:—

"I attribute the origin of this case to the use of the water at Jerusalem, and consider ourselves fortunate in having escaped with only one case of enteric fever among the seventy-two persons visiting it. Without the walls of Jerusalem the water appears to be very good, but inside it is received into vast tanks and reservoirs beneath the Harem area, and elsewhere. These, from what I saw in the excavations recently executed by the Palestine Exploration Society, are entirely without protection from receiving a large proportion of the sewage of the city, in some cases without even the slightest filtration through earth, or other obstacle. At the Fountain of Siloam, and Pool of Siloam, the water distinctly tasted like soap-suds, brought down by the water from the baths, &c., close to the Temple enclosure."—*Lancet.*

***Treatment of Chronic Constipation.***

The Rev. David Bell, M.D., of Yorkshire, England, urges the advisability of combining tonics with aperients in cases of irregular and slow action of the intestinal canal, and states that he has for many years found the following prescription uniformly efficacious in overcoming habitual constipation:

| | |
|---|---|
| ℞. Aloes Socotrin., Ext. Hyosciami, - - - | āā gr. xii; |
| Quinin. Disulph. - - - - - - - | gr. vi; |
| Ferri Sulph. - - - - - - - | gr. iv; |

M. bene et fiat massa in pilulas xii æquales dividenda.

One of these pills, taken in the afternoon from four to six o'clock, will cause a formed natural motion the next morning, without pain. The proportion of aloes may in some cases be reduced, to assimilate its action to that of nature. Its supposed influence in causing or aggravating hæmorrhoids has not been found by the author in the use of the above combination.—*Med. Gazette.*

***Treatment of Syphilitic Swellings.***

Prof. Sigmund recommends punctures by an exploring trocar, or by a Pravaz's syringe. The puncture must be performed once or twice a day, followed by an injection of linseed oil with carbolic acid, or by a compress bandage. After the puncture the pus can also be evacuated by slight pressure. In regard to the general treatment, the author mentions the hypodermic injection of chloride of silver. The solution contains four grains to an ounce of water. At first, 12 drops are injected twice a day. The groin and the infra-clavicular region should not be chosen as sites for injection. The cure is rapid, but not sure. In suppurating buboes, S. applies a solution of carbolic acid one part, with linseed oil four parts and lime 32 parts, after Lister. It is useless in periadenitis. It requires the same length of time as any other method, but in cases of large cavities it manifests its good effects by diminishing the secretion and bringing up healthy granulations. —*Bericht d Wiener Krankenhauser.—Indiana Jour.*

***Employment of Phosphorus in Psoriasis.***

Of late, several experiments with phosphorus have been made in the various hospitals of Paris. It has been tried in some cases to which it had not until now been applied. Amongst others, we may just mention the employment of phosphide of zinc in locomotor ataxy and phosphorus in psoriasis. This last trial is due to Prof. Hardy, the well-known dermatologist. M. Hardy had previously employed the substance in similar cases, and had renounced its use; but during the last few months he has resumed his researches. There are now five patients in his wards—three female and two male—subjected to this mode of treatment. One of the female patients, aged sixteen, and affected with general psoriasis, has taken every day since Dec. 16th a teaspoonful of the following compound—oil, 150 grammes; phosphorus, 10 centigrammes; and has been rubbed every night with a pomade composed of—hog's lard, 100 grammes; phosphorus, 1 gramme. Under the influence of this treatment the scales have completely disappeared. No unusual symptom has been observed.—*Lancet.*

***Effect of Medicines on Temperature.***

Mr. F. J. Mavor, in a note to the *Lancet*, asserts, as the result of numerous experiments, that atropine, morphine, strychnine, opium, belladonna, digitalis, chloral hydrate, nitric and muriatic acids, nitrate and carbonate of potassa, ammonia, sulphate of magnesia, iron, and aloes, all raise the temperature both in health and disease. If so, it would perhaps be well to overhaul our list of antiphlogistics for further experimentation.

### *Lithotomy.*

Mr. Holmes Coote says (*Lancet*, May 21st, 1870) that "lithotomy, properly performed, is not in itself so serious an operation as some authors have made it. The last twenty-two cases performed at St. Bartholomew's Hospital have been without exception successful. In unfortunately fatal cases the cause of death is not hæmorrhage, or very rarely so. I cannot recall a case of death from hæmorrhage at St. Bartholomew's Hospital, during the whole period that I have been attached to that institution. Neither is extravasation of urine a common cause of death with good operators. But when the kidneys have become diseased, the patient's power of recovery seems wonderfully diminished, and inflammation of the peritoneum is an event which the surgeon justly holds in dread."

### *Croup and Lactic Acid.*

Dr. A. Weber states that since he commenced using lactic acid in croup, he has had no need to operate, and has not lost a case. Every half hour from fifteen to twenty drops of the acid to half an ounce of water are to be inhaled through an apparatus for that purpose. After the dyspnœa has been diminished, the dose is to be lessened to five drops every hour.

### *The Lady Doctor.*

Yesterday I went to the ball,
  I'll tell you now the issue,
Dancing is healthy for us all,
  (It renovates the tissue).

My partner for the first quadrille
  Spoke soft of "lovers' perjury;"
I'd never heard of that disease
  In all the range of surgery.

He swore that he was mad with love,
  In accents quite theatrical;
I hailed him as an object fit
  For studies psychiatrical.

He swore his heart was breaking fast;
  I begg'd he'd try a blister,
But said that broken hearts to set
  Would puzzle e'en a Lister!

He said that he should pine away,
  In accents melancholic;
I recommended 'gainst decay,
  The acid called carbolic.

He left me, but consoled himself
  With soda and with sherry—
He knew, no doubt, the first alone
  Is deleterious, very.

The next with whom I danced had such
  A well developed head, sir,
I wished that, as a legacy,
  He'd leave it me when dead, sir.

'Tis true he couldn't waltz—indeed,
  He floundered like a grampus—
But then his brains!—I'm sure he had
  A monstrous hippocampus!

A student medical was he,
  A man of humor comical;
O how he'd laugh when'er he made
  A *bon mot* anatomical!

And so we left the dance and band,
  I'm sure you'll own we'd gumption;
The galop sole of which we spoke
  Was galloping consumption.

The waltzers at the *trois temps* danc'd
  As they'd go on forever—
The only *trois temps* we thought on
  Was tertiary fever.

But here I'll pause; I'll only add,
  To gratify my tutors,
I practiced for anatomy
  By "cutting up" my suitors.

—*Eclectic Journal.*

